# A Glyphosate-Based Formulation but Not Glyphosate Alone Alters Human Placental Integrity

**DOI:** 10.3390/toxics9090220

**Published:** 2021-09-13

**Authors:** Christelle Simasotchi, Audrey Chissey, Gérald Jungers, Thierry Fournier, Gilles-Eric Seralini, Sophie Gil

**Affiliations:** 1 Faculté de Pharmacie, Université de Paris, INSERM, U1139, 3PHM, 4 Avenue de l’Observatoire, F-75006 Paris, France; christelle.simasotchi@premup.org (C.S.); audrey.chissey@parisdescartes.fr (A.C.); thierry.fournier@parisdescartes.fr (T.F.); sophie.gil@parisdescartes.fr (S.G.); 2 Network on Risks, Quality and Sustainable Development, MRSH, Faculty of Sciences, University of Caen Normandy, Esplanade de la Paix, F-14032 Caen, France; gerald.jungers@ac-caen.fr

**Keywords:** glyphosate, roundup, formulants, toxicity, placenta, human

## Abstract

Glyphosate (G)-based herbicidal formulations, such as the most commonly used one, Roundup (R), are major pesticides used worldwide on food and feed. Pregnant women may be frequently exposed to R compounds. These are composed of G, which is declared as the active principle, and other products contained in formulations, named formulants, which have been declared as inerts and diluents by the manufacturers. These formulants have, in fact, been demonstrated to be much more toxic than G, in particular to placental and embryonic human cells. In this work, we thus compared the effect of G and a GT+ formulation named R, using placental perfusion ex vivo. R, but not G alone, was demonstrated to alter the placental permeability of a known small model molecule, antipyrine. Similar results were observed for the fetal venous flow rate. The transfer of G alone increases with time, but is significantly decreased in presence of its formulants. The perfusion of R provokes a destruction of fetal vessels, as demonstrated by immunohistochemistry. Formulants obviously alter the fetal-placental circulation and placental integrity according to time of exposure. Therefore, G does not appear to be the main toxic agent of R. Formulants, although undeclared, include polyoxyethanolamines, PAHs, or heavy metals, and may be responsible for this toxicity. These compounds are also present in other pesticides. The progressive blood flow reduction due to the toxic compounds of formulations may diminish the nutrient supply to the fetus, alter the development, and may enhance the poisoning effects. Although these are preliminary results, they could at least partially explain some adverse pregnancy outcomes in mothers exposed to pesticides or other environmental pollutants. The debate on glyphosate alone is proven insufficient for the understanding of the toxicity.

## 1. Introduction

**Figure 1:**
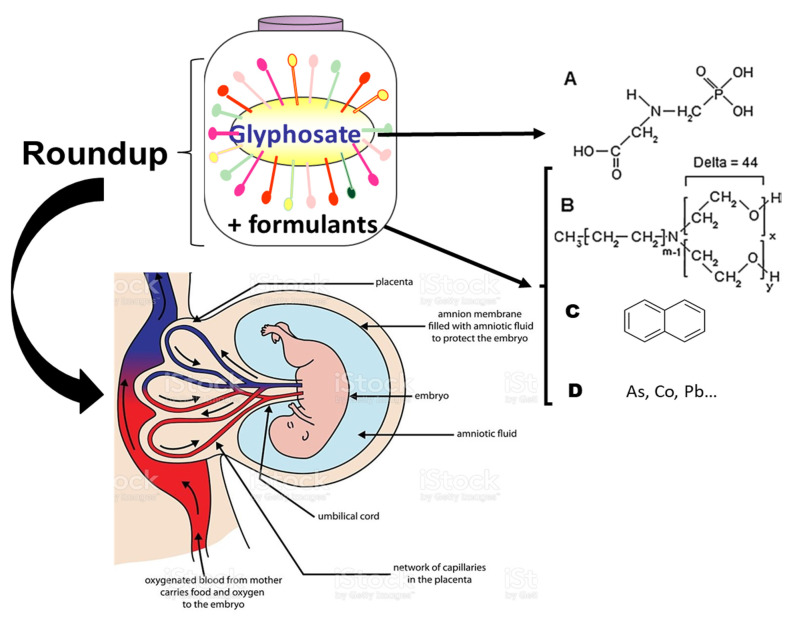
Roundup composition containing glyphosate and its formulants tested through human placenta. Roundup R is composed of a declared active principle glyphosate G (**A**) and other products (formulants) in the commercialised herbicide product, and evidenced more recently, such as polyethoxylated alkylamines POEA (**B**), naphtalene (**C**), and heavy metals (**D**). The transfer of R (with formulants) and G (without formulants) through the placenta ex vivo are studied here.

Glyphosate-based herbicidal formulations (GBH), such as the major one known as Roundup (R), are the most commonly used pesticides worldwide [1]. They are used on large areas of crops that are genetically modified (GM) to be tolerant to R (ISAAA, 2019). Their residues are thus consumed by intensively farmed animals fed with GM soy or corn [2]. The chemicals from R also more directly enter the human food chain, since wheat and or other cereal crops (e.g., those used for beer) are treated with it before harvest [3]. These contaminate rivers and other water sources [4]. G as a marker has even been measured in human urine and tissues [5]. It has been decreased with its metabolite AMPA in adults and children urine by an organic diet [6]. Its formulants are also measured in human urine [7]. Pregnant women may thus be frequently exposed to R compounds. These are composed of G, declared as the active principle, and other products of formulations, known as formulants (Figure 1), which have been declared as inerts and diluents by the manufacturers but have in fact been demonstrated to be much more toxic than G [8,9], in particular to placental and embryonic human cells [10]. At lower non-cytotoxic levels, R formulations have been shown to be endocrine disrupters [11], to a much greater extent than G alone.

These formulants in different R formulations have been carefully analyzed and include a family of polyethoxylated alkylamines (POEA), as well as detergents, oxidized petroleum residues, and heavy metals, such as arsenic, cobalt, chromium, nickel, and lead [12] (Figure 1). This list is not exhaustive, but the full list is unknown, since the formulants have been classified as inerts or not declared by the manufacturers. Polycyclic aromatic hydrocarbons (PAHs) have even been recently found in R formulations which are not G-based, but instead contain acetic or pelargonic acids [13].

Since all the above-mentioned chemicals are themselves toxic to human placental and embryonic cells, as previously underlined [10], and also in vivo to mammals [14]), in this work, we investigated the differential toxicity between G and R directly to the human placenta, and the consequent placental transfer through this natural barrier ex vivo.

An increasing number of birth defects [15,16], adverse pregnancy outcomes [17,18], and other diseases (such as neurodevelopmental disorders [19] and childhood cancers [20]), are reported in the world and are being linked to environmental pollution. Moreover, the debate on R in the scientific literature has been mostly centered on G. We thus compared the effect of G and R on the most sensitive interface between mother and child as a preliminary approach.

## 2. Materials and Methods

### 2.1. Chemicals and Chemical Analyses

As a model for a representative G formulation, R GT+ containing 45% G (450 g/L), market authorization 2020448 from Monsanto Company has been used and diluted in phosphate-buffered saline (PBS) at 1 ppm. G (N-phosphonomethyl glycine, G, CAS 1071-83-6) was obtained from Sigma–Aldrich, Saint Quentin Fallavier, France. G is assayed by liquid chromatography followed by double mass spectrometry [21], together with its main metabolite aminomethyl phosphonic acid (AMPA). In R, formulants POEA and metals have been previously measured [22] and naphtalene as a marker for PAHs has been evidenced in R in this study via the DIN 38407-39 method, using gas chromatography and mass spectrometry [13].

### 2.2. Human Tissues and Ethics

Full-term placentas (37–40 weeks of gestation) from uncomplicated pregnancies were quickly collected after vaginal delivery or caesarean section from Port-Royal Obstetric Department (Paris, France) since the time to full perfusion (mother and fetus) should not exceed 30 min. Women did not receive any medication during pregnancy, except for epidural analgesia or oxytocin during labor, if necessary. The patients signed a consent to obtain the placenta for experiments (CPP Paris Ile-de-France 3, N-18-05, Paris, France).

### 2.3. Placental Perfusion

Collected placentas (19, 3–4 at least per experiment) were perfused in a double circuit according to a validated method as previously described [23]. The solutions were put in Earle medium with 25 g/L of bovine serum albumin (Euromedex, Souffelweyersheim, France). The pH was adjusted to 7.4 ± 0.1 and 7.2 ± 0.1 for maternal and fetal compartments as described. Perfusions were started immediately after delivery (within 20 min). After a visual examination the vascular integrity was confirmed, a distal branch of a fetal artery and its associated vein supplying a selected cotyledon were cannulated. The flow rate of 6 mL/min was established in the fetal circulation. This ensured a balance between both arterial and fetal venous flows. The placentas possibly showing vascular leakages were not used. The chosen cotyledon whitened progressively during perfusion from the maternal side, and this evidenced the working system. A wash of 10 min preceded the insertion of two catheters into the intervillous space, in the maternal side. The maternal circulation was 12 mL/min. The perfusion was checked by pressure in the fetal compartment. Two molecules were used to validate placental perfusion, i.e., antipyrine (A) as a positive control and FITC-Dextran as a negative control. After a 5 min wash, A [24] was perfused because it is a small molecule which crosses the placental barrier without tissue accumulation. Usually, A (final concentration 20 mg/L, CAS 60-80-0, from Sigma-Aldrich, Saint Quentin Fallavier, France) is used as a marker to validate the model of placental permeability; it shows the overlap between the maternal and fetal circulations. Then FITC-Dextran was used as a negative control (final concentration 660 μg/mL, CAS 60842-46-8, from Sigma-Aldrich, Saint Quentin Fallavier, France). FITC-Dextran is a large molecule that does not pass through the placental barrier and thus proves its integrity. G at 1 ppm alone, or in R formulation, was subsequently added in the same compartment. The perfusion maximal duration was 240 min. Samples from maternal circulation and venous fetal circulation were collected each 30 min following A transfer, and at 5120, and 240 min in order to monitor G concentrations. At the end of perfusion, all the samples were stored at −80 °C until analysis.

### 2.4. Calculation of Placental Parameters

Fetal venous flow rates were standardized to the value at the beginning of the perfusion. At the equilibrium, the fetal circulation was established at a flow rate of 6 mL/min to ensure a balance between arterial and fetal venous flow (at this step, the value of the fetal venous flow rate corresponds to 100%). Standard parameters were calculated according to the formulas of Challier [25]. Placental transfer was estimated for two parameters: the FTRs of A and of G. The FTR was calculated as follows: FTR = (Cf/Cm) × 100, where Cf is the venous fetal concentration of a molecule and Cm is the maternal concentration of the same molecule. The result is given with a percentage. For normal healthy placentas, a maximum value of FTR was reached after 15 min of perfusion. This value must be stable until the end of the perfusion. FTR of A had to be over 20% [25]. Otherwise, the placenta was not used in the study.

### 2.5. Immunohistochemistry

Immunohistochemistry was performed on the placental cotyledon used for perfusion. At the end of each perfusion, the cotyledon was fixed in 4% PFA overnight at 4 °C, washed in PBS 1X. About 5 mm^3^ of tissue was embedded in 4% (*w*/*v*) agarose (Electran, low melting ref 444153H, VWR, Rosny-sous-Bois, France) and tissue sections (150 µm and 200 µm) were realised using a vibratom. Sections were permeabilized with 1% Triton X-100 for one hour and then saturated with 3% IgG-free BSA, 7% goat serum, human IgG (final concentration: 12.5 μg/mL), 0.01% Triton X100 for 4 h at room temperature. Tissue sections were incubated under agitation overnight at 4 °C, with anti-CD31 antibody at 2 µg/mL (M0823, Dako, Glostrup, Denmark) for endothelial cells labelling, anti-CK7 antibody at 1 µg/mL (SAB4501652, Sigma, Saint-Quentin-Fallavier, France) for trophoblast labelling in 1% PBS BSA. After washing in 0.05% PBS Triton, sections were incubated for 2 h under agitation at room temperature with the appropriate fluorochrome-conjugated secondary antibody (1:500, Alexa Fluor 488 or 546 in PBS BSA 1%). Nuclei were stained with DAPI (0.2 µg/mL) and the sections mounted with Fluoromount-G mounting fluid (#FP-483331, Interchim, Montluçon, France). Acquisitions were made with a Leica SP8 confocal microscope and images analysed with Leica software (Nanterre, France).

### 2.6. Statistics

Statistical analyses were performed using GraphPad Prism 9.1 (GraphPad Software, San Diego, California, USA). All results are expressed as means ± standard errors of the means (SEM). Data were analyzed for significant differences using one-way analysis of variance (ANOVA), followed by Friedman test for multiple comparisons. Relationships between variables were assessed using the Pearson correlation coefficient ρ. A two-sided *p*-value < 0.05 was considered statistically significant.

## 3. Results

### 3.1. Transplacental Transfer of A and Fetal Venous Flow in Presence of G Alone or with Its Formulants (R)

**Figure 2:**
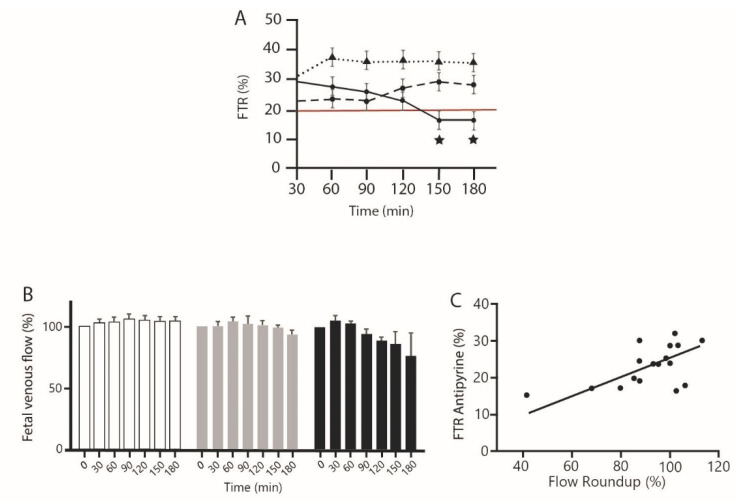
Fetal transfer rate and fetal venous flow of antipyrine in presence of glyphosate alone or with its formulants. (**A**) Effect of 1 ppm G alone or in R compared to antipyrine fetal transfer rate (FTR). ★ Significant differences, for 150 min *p* < 0.04, for 180 min *p* < 0.02. Small dots: control, dashed line: G alone, bold curve: R. Red line, minimal normal FTR. (**B**) Effect of 1 ppm G alone or in R on fetal venous flow during placental perfusion. Control is in white bars, grey bars are for G alone, R treatments correspond to black bars. (**C**) Correlation between antipyrine fetal transfer rate and fetal venous flow for R at all times. Values given are raw data. Significant correlation *p* < 0.0102; *r* = 0.58.

Two control parameters were used to validate the perfusion experiments: a positive control with A that should evidence at least a transfer rate of 20% [25], as was the case here, and a negative control with FITC-Dextran, that does not transfer when there is placental integrity. No FITC-Dextran was detected in the fetal circulation, whatever the treatment. The experiences were validated. The three placentas were successfully perfused for each condition: A control alone, or together with G alone at 1 ppm, or with the same concentration of 1 ppm of R (or GBH) in total, i.e., G in mixture with its formulants, declared as inert diluents by the manufacturer (Figure 2). The study of the mean transfer rate of A is described in Figure 2A according to the three treatments. For control placentas and G alone, we showed that a maximum value of FTR was reached and remained stable during all the perfusions. The FTR calculated for A in the presence of 1 ppm of G was always below the control but not statistically different from control.

For placentas perfused with GBH also called R here, we observed that the FTR decreased slightly from 30 to 120 min. Then, the FTR decreased significantly to reach a value below the threshold of 20% from 150 min. Similar results were observed for the fetal venous flow rate. It decreased in the same time during the perfusion with G in R (Figure 2B) and correlated positively (Figure 2C, *r* = 0.56) with the A FTR, indicating a loss of placental function through venal flow decrease to the fetus.

### 3.2. G Transplacental Transfer Rate Alone or in Its Formulants (R) in Comparison to A

**Figure 3:**
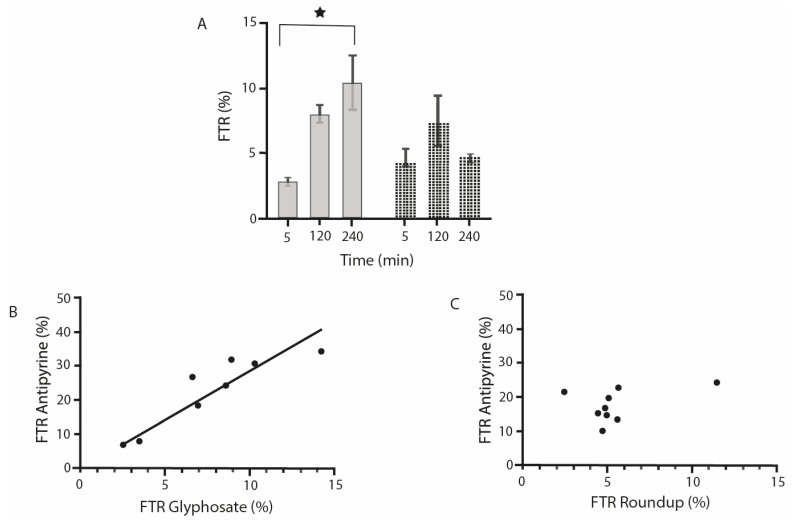
Fetal transfer rate of G alone or in its formulants (i.e., R) and correlations with antipyrine. (**A**) G + AMPAtransplacental transfer rate. G is measured by LC-MS-MS, together with its metabolite AMPA; values are given as mean ± SEM. ★ Significant difference *p* = 0.049. In grey: G alone; in dotted bars: G with formulants corresponding to R. (**B**) Correlation between A transfer rate and G transfer rate. For all times compiled *r* = 0.9039; *p* = 0.0021. (**C**) No real correlation between A transfer rate and R transfer rate. For all times compiled *r* = 0.403; *p* = 0.2811.

We observe in Figure 3A that after 5 min, the transfer of G alone or associated with its formulants (R) is already detectable. After 120 min of perfusion, the transfer of G alone or associated with its formulants (R) is similar. After 240 min the transfer of G alone is significantly higher (approximately five-fold) than after 5 min (*p* = 0.049). However, after 240 min, the transfer of G is decreased in the presence of its formulants. In Figure 3B, a very significant correlation is shown between the transfer rates of G and A, whatever the time of perfusion. Figure 3C demonstrates that there is no such correlation between the transfer of G in formulants and A.

### 3.3. Placental Histology after 240 min Perfusion of G or R

**Figure 4:**
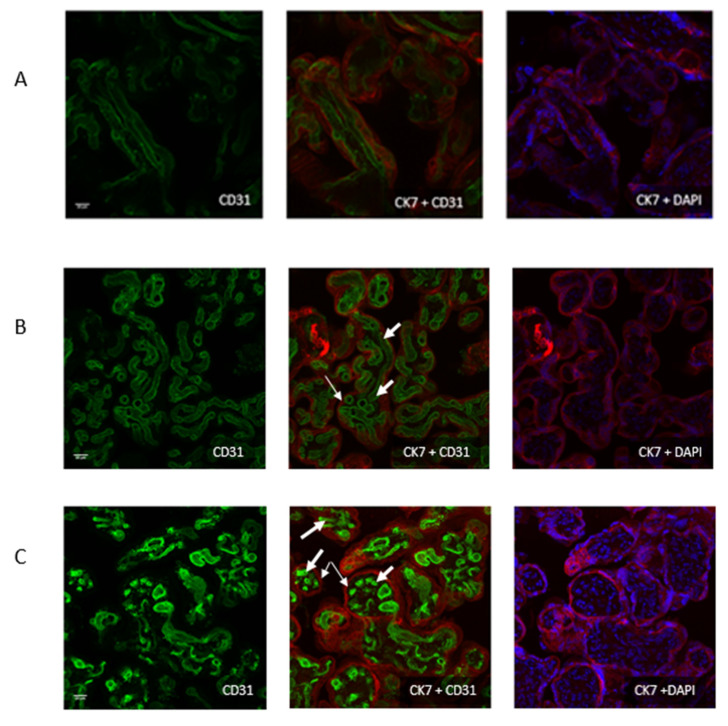
Acquisitions were performed on 240 min-perfused placentas with a Leica SP8 confocal microscope (40X/1.3) in immersion. Anti-CD31 antibodies were used for endothelial cells and thus cytotrophoblast labelling (thin arrows), anti-CK7 antibodies for syncytiotrophoblast labelling (thick arrows), and nuclei were stained with DAPI. (**A**) control, (**B**) after G perfusion, (**C**) after R perfusion.

In control placentas, as well as the ones treated with G perfusions (Figure 4A,B), there was no visible alteration of fetal vessels after 240 min perfusion. A normal structure was observed. This was demonstrated on both cytotrophoblast and syncytiotrophoblast aspects, as evidenced by differential staining. On Figure 4C, a destruction of fetal vessels was noticed, showing the effect of R perfusion. The presence of formulants was the only difference. Thus, the formulants alter the placental circulation and integrity, according to this time of exposure.

## 4. Discussion

Using the perfusion of an isolated cotyledon of human placenta, we describe for the first time a comparative study on G and R placental transfer from the mother to the fetus. Glyphosate transfer was already studied [26]. These authors proposed to use data collected during experiments to build a predictive model for transfer of various xenobiotics. They chose molecules with low molecular weights (caffeine, benzoic acid, glyphosate) with different properties. Caffeine freely diffused, while glyphosate crossed partially the placental barrier. The amount of drug reaching the fetus depends not only on physicochemical characteristics of molecules (e.g., size, protein binding), but also maternal pharmacokinetics parameters and placental parameters, varying according to the term of pregnancy. We used two control markers, A and FITC-Dextran. The addition of A into the maternal compartment can be used to validate the model of placental permeablility. Without protein binding and cellular accumulation, A transfer rate depends only on fetal and maternal flows, which should be constant during normal perfusion.

The addition of FITC-Dextran as a negative control can be used to ensure that placental tissue is preserved without transcellular passage of this large molecular marker.

Since G alone, as a comparably small sized molecule, did not modify statistically the normal transfer of A, we can conclude that G at a relatively high level (1 ppm), found at ppb levels in humans [5], is not the main toxic agent on this parameter. However, when G is perfused with its formulants used in the commercial mixture called R, the fetal transfer of small molecules such as A is affected from 150 min. Usually, the balance of flows in the fetal circuit was controlled throughout the perfusion procedure. Given the fact that the fetal artery flow was known (induced by the peristaltic pump), if a difference was noticed between the fetal artery and the fetal vein, it was possible to conclude that the balance of flows was incorrect (leading to a failure of placental transfer). In this case, this may represent a fetal progressive deprivation from nutrients and essential life factors. Polyoxyethanolamines, PAHs, or heavy metals may thus be responsible for the toxicity examined in this study. This deleterious effect may begin slightly from 1 h but become significant after 2.5 h. We also demonstrated in vitro that fetal and embryonic human cells were sensitive to these pesticide formulants after a few hours [8,10]. This was demonstrated by an inhibition of mitochondrial succinate dehydrogenase (SDHI), damaging cell respiration, membrane and nuclei integrities, promotion of apoptosis, and, at lower levels, endocrine disruption. In vivo, naphthalene, for instance, can cause hemolytic anemia, respiratory failure, methemoglobinemia, and hepatic and renal injuries in newborn infants, as well as oxidative stress [27]. POEA may also by itself induce adverse mammalian reproductive changes [28,29]. Heavy metals can have similar effects, as is widely known. Maternal exposure to glyphosate formulants that are also used for other pesticides [12,13] may be chronic during pregnancy. We demonstrate here that the placental permeability, even for small molecules, is disrupted at the ppm level in a few hours, but we previously demonstrated that this represents a model of the chronic ppb effects of the formulants that become visible in vivo after a few weeks or months [14]. All these formulants may present detersive, disruptive, and destructive effects on placental membranes. The fetal venous flow rate is consequently diminished afterwards, as demonstrated here. Fetal circulation and respiration can be affected by these impacts.

In the following results, we clearly observe that the transfer of G alone is possible and may increase with time, possibly resulting in G bioaccumulating in the fetal compartment. Then toxic effects may not be excluded after a chronic exposure, even without the formulants. However, in this work, we have no evidence of G toxic effects alone. By contrast, R does not behave as G. The presence of the formulants may provoke a specific degradation of the human placenta. The permeability is highly altered. A venal collapse was checked histologically for this reason. It was confirmed in our present results. The progressive blood flow reduction due to the toxic compounds of formulations may diminish the nutrient supply to the fetus and enhance the poisoning of the embryo. Depending on the time and duration of exposure, this may impact or modify development of the fetus during pregnancy, including organ formation. These impacts may potentially cause the malformation of a newborn or even a fetal death.

The mechanisms of exchanges between mother and fetus appear to be very different between a first trimester placenta and a term placenta with important structural differences. In particular its vascularization [30]. In addition, carrier expression profiles vary during pregnancy [31]. These differences in the expression of the transporters in addition to the structural differences between the first trimester and term do not make it possible to transpose the results observed at term for a placenta of early pregnancy. However, our results are characteristic of the last trimester. During the first and second trimesters, the placenta could be more sensitive to xenobiotics, and this has to be studied if possible.

Although these are preliminary results, they could at least partially explain some pregnancy outcomes in mothers exposed to pesticides or other environmental pollutants. Since these formulants are undeclared, this may be a socially relevant issue for the assessment of the toxicity of pesticides. Based on our results, the debate on glyphosate alone is insufficient for that scientific understanding.

## Abbreviations

A: Antipyrine; AMPA: aminomethyl phosphonic acid; BSA: bovine serum albumin; FITC: Fluoresceine isothiocyanate; FTR: Fetal transfer rate; G: Glyphosate; GBH: Glyphosate-based herbicidal formulations, also named R in this work; GM: genetically modified; PAH: polycyclic aromatic hydrocarbons; PBS: phosphate-buffered saline; PFA: perfluoroalkoxy; POEA: polyethoxylated alkylamines; R: Roundup; SDHI: succinate dehydrogenase inhibition.
